# Cili (*Rosa roxburghii* Tratt.) Fruit and Its Co-Culture Fermentation with *Bacillus subtilis* and *Lactiplantibacillus plantarum* Improves the Nutritional, Functional and Bioactive Quality of White–Black Wheat Composite Bread

**DOI:** 10.3390/foods15203705

**Published:** 2026-10-17

**Authors:** Angelo Uriho, Jacob Ojobi Omedi, Binle Zhang, Liuping Fan, Jianxian Zheng, Yongqing Zeng, Shuning Zhang, Li Liang, Yan Xu, Weining Huang

**Affiliations:** 1 State Key Laboratory of Food Science and Resources, Laboratory of Baking and Fermentation Science, Cereals/Sourdough and Nutritional Functionality Research, School of Food Science and Technology, Jiangnan University, Wuxi 214122, China; urihoa@gmail.com (A.U.); omedij@yahoo.com (J.O.O.); zbl_500@163.com (B.Z.); fanliuping@jiangnan.edu.cn (L.F.); shuningzhang@jiangnan.edu.cn (S.Z.); liliang@jiangnan.edu.cn (L.L.); 2 Department of Food Engineering, Zhangzhou Institute of Technology, Zhangzhou 363000, China; 3 College of Food and Bioengineering, South China University of Technology and Guangzhou Institute of Food Industry, Guangzhou 510000, China; fejxzhen@scut.edu.cn (J.Z.); yongqingzeng2026@163.com (Y.Z.); 4 Key Laboratory of Industrial Biotechnology of Ministry of Education, School of Biotechnology, Jiangnan University, Wuxi 214122, China; yxu@jiangnan.edu.cn

**Keywords:** γ-aminobutyric acid, whole pigmented grain, functional bread, phytochemical compounds, soluble dietary fiber

## Abstract

Whole black wheat increases the dietary fiber and anthocyanin content of bread, but reduces loaf-specific volume and increases crumb firmness. This study evaluated Cili (*Rosa roxburghii* Tratt.) fruit co-culture fermented with *Bacillus subtilis* and *Lactiplantibacillus plantarum*, as an ingredient (30%, flour weight) in white wheat (BsLp) and white–black wheat (50:50) (WWBW-BsLp) composite bread, and compared with the breads without Cili (white wheat: WB; white–black wheat: WWBW). In dough, β-glucosidase, feruloyl esterase, and α-amylase activities were elevated in BsLp and WWBW-BsLp after proofing. In bread, compared with WB, WWBW-BsLp contained more GABA (312.2 vs. 5.7 mg/kg DW), total phenolics (484.8 vs. 70.7 mg GAE/100 g DW), total flavonoids (58.0 vs. 17.0 mg QE/100 g DW), and total dietary fiber (9.46 vs. 3.01 g/100 g DW), including more soluble fiber (2.29 vs. 0.30 g/100 g DW) (all *p* < 0.05). DPPH and ABTS radical-scavenging activities increased from 11.9% to 72.9% and from 28.8% to 80.0%, respectively. Compared with WWBW, inclusion of fermented Cili reduced crumb firmness (410 vs. 493 g; *p* < 0.05) but did not significantly change specific volume (4.17 vs. 3.94 mL/g). Co-fermented Cili is thus a promising ingredient for increasing GABA, phenolics, and soluble fiber in composite bread.

## 1. Introduction

Bread is one of the most widely eaten staple foods globally, making it an effective way to deliver health-promoting ingredients to a large audience. Consumer preferences have shifted from viewing bread as merely an energy source to favoring functional bakery products rich in dietary fiber and bioactive phytochemicals, which are associated with the prevention of diet-related non-communicable diseases, including obesity, type 2 diabetes, and cardiovascular disorders [1,2]. However, refined white wheat bread lacks the bran and germ which contain most of the fiber, minerals, and phenolic compounds, thereby limiting its functional benefits. In cereal science, a key challenge is reformulating bread to include these beneficial components without affecting its volume, texture, and flavor, attributes crucial for consumer acceptance.

Pigmented wheats, especially black wheat, have become promising ingredients for this purpose. Developed by combining the anthocyanin traits of purple and blue wheat, black wheat deposits anthocyanins in the pericarp and aleurone layers and contains higher levels of dietary fiber, phenolic acids, flavonoids, and minerals compared to traditional wheat, resulting in greater antioxidant activity [1,3]. Diets rich in pigmented whole wheat are associated with better lipid profiles, glucose regulation, and oxidative balance. Since bakery products are among the most widely eaten foods worldwide, adding anthocyanins can boost their health benefits. However, using whole black wheat in bread often dilutes and disrupts the gluten network because bran particles and insoluble fibers compete for water, typically resulting in a smaller loaf volume, a firmer crumb, and a darker color [2,4].

Cili (*Rosa roxburghii* Tratt.), a spiny wild fruit from southwestern China’s Yunnan–Guizhou plateau, is a highly rich yet underused source of bioactive compounds. Renowned for its high vitamin C content, it also contains numerous polyphenols, flavonoids, triterpenoids, superoxide dismutase, and polysaccharides, all of which exert potent antioxidant, anti-inflammatory, and metabolic-regulating effects [5,6]. Its glutamate-rich composition makes it ideal for biosynthesizing γ-aminobutyric acid (GABA). However, the fruit’s sourness and astringency limit direct consumption, so most of it is processed into juice or discarded as pomace. As a potential food ingredient, fermentation of Cili reduced its sourness and astringency and increased its bioactive content. Fermenting Cili juice with *L. plantarum* increased its phenolic content and antioxidant capacity [7]. Tandem fermentation of a Cili–coix seed beverage with *Trametes versicolor* and *L. plantarum* increased GABA and polysaccharides, reduced bitterness, and retained over 90% of vitamin C [8]. In cereal products, adding 10% *Ganoderma lucidum*-fermented Cili pomace to noodles lowered starch digestibility and improved sensory properties [7]. Moreover, the co-culture fermentation of Cili with *B. subtilis* and GABA-producing lactic acid bacteria enriched GABA and increased hydrolytic enzyme activities in the fruit substrate [9]. Adding this fermented fruit to refined wheat bread increased GABA and phenolic contents while keeping loaf quality and sensory acceptance close to those of the wheat control [10]. However, all of these studies used refined flours. Whole pigmented wheat behaves differently in dough: its bran dilutes the gluten network, competes for water, and contains bound phenolics and anthocyanins [1,2]. Few known studies have explored the effect of Cili fruit and whole black wheat flour in a dough–bread composite system.

Microbial fermentation is an eco-friendly and effective way to release bound bioactives and create new functional metabolites. *B. subtilis* secretes extracellular hydrolases, including β-glucosidase, feruloyl esterase, xylanase, amylases, and proteases, which cleave the glycosidic and ester bonds linking phenolic compounds to cell-wall polysaccharides, thereby increasing the release of free phenolics [11,12]. *L. plantarum* primarily acidifies the substrate by producing lactic and other organic acids and converts glutamate to GABA via glutamate decarboxylase (GAD) [13,14]. Some strains also exhibit β-glucosidase and phenolic esterase activities [15]. Co-culturing the two species may therefore combine enzymatic phenolic release by *B. subtilis* with acidification and GABA synthesis by *L. plantarum* in a single ingredient [15,16].

Recently, we screened GABA-producing LAB and optimized their co-culture fermentation with *B. subtilis* on a Cili fruit substrate, confirming GABA enrichment, along with increased enzyme activities and bioactive properties in the fermented substrate [9]. However, it remains uncertain whether this co-culture fermented Cili fruit can be used effectively in bread, particularly whether it can mitigate the quality issues associated with whole black wheat while enhancing its nutritional and functional benefits. Therefore, the aim of this study was to evaluate the effects of Cili fruit puree co-fermented with *B. subtilis* and *L. plantarum* on the dough physicochemical and rheology properties, and the nutritional, bioactive, in vitro functional, and quality of white wheat–whole black wheat composite bread.

## 2. Materials and Methods

### 2.1. Materials

Sun-dried Cili (*Rosa roxburghii* Tratt.) fruits were purchased from Guiding County Zhou Xigeng Special Food (Duyun city, Guizhou, China). Commercial refined white wheat bread flour (energy: 1490 kJ/100 g; protein: 12.7 g/100 g; carbohydrates: 71.9 g/100 g; saturated fats: 1.9 g/100 g; sugar: 1.9 g/100 g; sodium: 0 mg/100 g) was purchased from Xinxiang Liangrun Whole Grain Food Co., Ltd. (Xinxiang city, Henan, China), and organic whole black wheat flour (energy: 1580 kj/100 g; protein: 14.6 g/100 g; carbohydrates: 73.1 g/100 g; saturated fats: 2.4 g/100 g; sodium: 0 mg/100 g) was obtained from Xinjiang Nuobixin Agricultural Technology Co., Ltd. (Urumqi city, Xinjiang, China). Monosodium glutamate (MSG), γ-aminobutyric acid (GABA) and pyridoxal phosphate (PLP) were purchased from Shanghai Yuanye Biotechnology Co., Ltd. (Shanghai, China). *Bacillus subtilis* subsp. subtilis BNCC190341 was purchased from Beina Chuanglian Biotechnology Co., Ltd. (Xinyang city, Henan, China). *Lactiplantibacillus plantarum* (HWN19) was obtained from the Laboratory of Baking and Fermentation Science, Cereals/Sourdough and Ingredient Functionality Research, Jiangnan University.

### 2.2. Co-Culture Fermentation of Cili Fruit

Sundried whole Cili (*Rosa roxburghii* Tratt.) fruits (moisture content 12.98 ± 0.33%, determined by oven-drying at 105 °C to constant weight) were milled into powder using an electric stainless-steel grinder (DAMAI, Wuyi Haina Electric Appliance Co., Ltd., Jinhua city, Zhejiang, China), and passed through a 60-mesh (250 µm) sieve. The resulting powder was mixed with sterile distilled water at a powder-to-water ratio of 20:80 (*w*/*w*) by manual stirring for 5 min until a uniform puree was obtained. Co-culture fermentation with *B. subtilis* and the GABA-producing *L. plantarum* was performed as described by Uriho et al. [9]. Briefly, 100 g of Cili fruit puree was weighed on an electronic balance GC, JE2002, readability 0.01 g (Shanghai Puchun Measuring Instrument Co. Ltd., Shanghai, China) and placed into a 250 mL Erlenmeyer flask and co-cultured in a shaking incubator (HZL-F160, Taicang Shi Qiangle Shiyan Shebei Chang, Suzhou, Jiangsu, China) (150 rpm) at 37 °C. Based on conditions from a central composite design, fermentation proceeded in two stages: a first aerobic stage with a 3% *B. subtilis* starter (8 log CFU/g) for 24 h under breathable film, followed by a second anaerobic stage with a 3% *L. plantarum* starter (8 log CFU/g) for 48 h after sealing the containers. The independent variables were fermentation time, monosodium glutamate (MSG), and pyridoxal phosphate (PLP); the response variable was GABA content; and the optimum conditions were 1.32% MSG, 0.13 mM PLP, and 72 h fermentation time for *B. subtilis*-*L. plantarum* co-culture. The fermented puree for use as an ingredient in bread had moisture content of 81.68%, 4.86 for pH, 19.30 mL for TTA, and a cell count of 10.83 log CFU/mL (Table S1).

### 2.3. Bread Formulation and Preparation

To optimize the white wheat: whole black wheat flour ratio (0:100, 30:70, 50:50, 70:30), using specific volume, fiber and anthocyanin enrichment as the selection criteria at the preliminary trial, the 50:50 ratio was chosen and used in the subsequent bread formulation and preparation. The four bread formulations are presented in Table 1. Based on our previous study on refined white wheat bread [10], 90 g of co-fermented Cili fruit-based sourdough, equivalent to 30% of the flour weight, was used. Except butter, all the ingredients (including co-culture fermented Cili fruit) were first mixed in a stand kneader (SM-25, Sinmag Equipment Co., Ltd., Wuxi, China) at slow speed (135 rpm, 3 min), then at fast speed (270 rpm, 2 min). Butter was then added, followed by slow mixing (135 rpm, 3 min) and fast mixing (270 rpm, 2.5 min). The dough was rested (5 min), divided into 90 g pieces, rounded, and rested again (5 min), then shaped and panned. Proofing was carried out in a proofing cabinet (Sinmag Bakery Equipment Co., Ltd., Wuxi, China) at 38 ± 0.5 °C and 85% RH for 90 min, then baked (top 170 °C, bottom 210 °C; Sinmag Bakery Equipment, Wuxi) for 17 min. Loaves were cooled on a rack for 2 h to room temperature (25 ± 1 °C) before analysis.

### 2.4. Dough Analysis

#### 2.4.1. Dough pH and Total Titratable Acidity (TTA)

The pH and TTA were determined from a 10 g dough sample mixed with 90 mL of distilled water. After thorough mixing, the pH was measured with a pH meter (FE-20, Mettler Toledo Co., Shanghai, China), and the TTA was expressed as the volume of 0.1 M NaOH required to reach a final pH of 8.5 ± 0.2 [17].

#### 2.4.2. Dynamic Rheological Properties

Dough for rheological measurements was prepared according to the formulations in Table 1 (but without yeast) and the mixing procedure described in Section 2.3. For analysis, dough samples taken immediately after mixing were used. Dynamic rheology was measured on a Discovery Hybrid Rheometer (DHR-3, TA Instruments, New Castle, DE, USA) with a 40 mm parallel-plate geometry at a 1 mm gap, following Li et al. [2] with modifications. Dough was placed on the Peltier plate, the geometry was lowered to the gap, excess sample was trimmed, and the edge was sealed with silicone oil. Samples were equilibrated at 25 °C for 5 min. A strain sweep established the linear viscoelastic region, and 0.1% strain was selected. Frequency sweeps (0.1–10 Hz, 25 °C) recorded storage modulus (G′), loss modulus (G″), and loss tangent (tan δ). Measurements were run in triplicate.

#### 2.4.3. Enzyme Activities

Proofed dough (5 g) was combined with 25 mL of distilled water, homogenized for 20 min, centrifuged at 10,000× *g* for 15 min at 4 °C, using an Eppendorf Centrifuge 5804 R (Hamburg, Germany), and filtered through a 0.45 µm filter.

β-glucosidase activity was measured following [18]: 0.1 mL of supernatant was mixed with 1.8 mL of 0.05 M sodium acetate buffer (pH 5) and incubated at 37 °C for 5 min; then, 0.1 mL of 5 mM p-NPG was added and incubated for another 10 min at 37 °C. The reaction was halted with 1 mL of 1 M Na_2_CO_3_, and OD_400_ was measured against a β-glucosidase standard curve. One unit produced 1 µmol of p-NP per minute. Feruloyl esterase activity was determined using 1 mM p-nitrophenyl butyrate in 50 mM Tris buffer (pH 8), following Wei et al. [14]: 20 µL of extract was added to 980 µL of substrate, incubated at 37 °C for 5 min, and the released p-nitrophenol was measured at 410 nm. One unit hydrolyzes 1 µmol of substrate per minute at 37 °C and pH 8.0. α-amylase activity was assessed by the DNS method demonstrated by Wang et al. [15]: 0.1 mL of supernatant was mixed with 0.9 mL of 1% soluble starch in phosphate buffer and incubated at 75 °C for 10 min. DNS reagent was then added, the mixture was boiled for 10 min, and OD_540_ was measured. One unit of α-amylase activity was defined as the amount of enzyme required to release 1 mg of reducing sugar per minute. Activities were expressed per gram of dough.

#### 2.4.4. Dietary Fiber Composition

Total (TDF), soluble (SDF) and insoluble (IDF) dietary fiber were determined by the AOAC [19] enzymatic-gravimetric method (Official Method 991.43). Freeze-dried dough/bread (1.000 ± 0.005 g) was suspended in 40 mL MES-TRIS buffer (0.05 M, pH 8.2) and sequentially digested with heat-stable α-amylase (95–100 °C, 35 min), protease (60 °C, 30 min) and amyloglucosidase (60 °C, 30 min), with pH adjusted to 4.1–4.8 before amyloglucosidase. For IDF, digests were filtered through Celite-packed crucibles, and residues were washed with hot water, 95% ethanol, and acetone. For SDF, the filtrate was precipitated with four volumes of 95% ethanol (60 °C, 1 h) and washed with 78% ethanol, 95% ethanol, and acetone. Residues were dried (105 °C, overnight), weighed and corrected for protein (Kjeldahl, N × 6.25) and ash (525 °C, 5 h). The IDF, SDF, and TDF contents were calculated using the following equations:

(1) IDF or SDF (%) = [(R_1_ + R_2_)/2 − P − A]/[(M_1_ + M_2_)/2] × 100

(2) TDF (%) = IDF (%) + SDF (%)

where R_1_ and R_2_ are the residue weights (mg) of duplicate samples, P is the protein weight (mg) in the residue, A is the ash weight (mg) in the residue, and M_1_ and M_2_ are the sample weights (mg) of the duplicates. All measurements were performed in duplicate, and results were expressed as grams per 100 g of dough/bread on a dry-weight basis (mean ± standard deviation).

### 2.5. Nutritional Properties of Breads

#### 2.5.1. GABA Content

GABA content was determined using the method described by Yucui et al. [20], with some modifications. Freeze-dried bread (1 g) was extracted with 25 mL of 5% trichloroacetic acid, ultrasonicated for 20 min, left to stand for 2 h, and filtered. After centrifugation at 10,000× *g* for 10 min, 400 µL of the supernatant was analyzed for GABA by HPLC (HP1100, Agilent, Santa Clara, CA, USA) using an Agilent Hypersil ODS column (250 mm × 4.6 mm) maintained at 40 °C. The UV detector was operated at 338 nm from 0 to 28 min and at 262 nm from 25 to 31 min. Mobile phase A consisted of sodium acetate buffer (8 mg/mL, pH 7.20 ± 0.02) and 220 µL of triethylamine, with 5 mL of Tetralin. Mobile phase B consisted of sodium acetate buffer (8 mg/mL, pH 7.20 ± 0.02), methanol, and acetonitrile (1:2:2). Both mobile phases flowed at 1.0 mL/min.

#### 2.5.2. Total Anthocyanin Content

Anthocyanins were extracted with acidified methanol (85:15 methanol:1 M HCl) per Francavilla et al. [4]: Freeze-dried bread (1.00 g) was extracted with 10 mL acidified methanol, shaken for 30 min, centrifuged (11,000× *g*, 30 min), and made up to 10 mL. The absorbance of the clarified extract was measured at 535 nm using a UV-1800 (Shimadzu, Tokyo, Japan). The total anthocyanin content (TAC) was calculated using Equation (3):

(3) $$\mathrm{TAC}=(\frac{A}{\varepsilon })\times (\frac{V}{1000})\times (\mathrm{MW})\times (\frac{1}{\mathrm{SWT}})\times (10^{6})$$

where A is the absorbance measured at 535 nm, ε is the molar absorptivity (for cyanidin-3-glucoside, ε = 25,965 cm^−1^M^−1^), V is the total volume of anthocyanin extract (in mL), SWT is the sample weight, and MW is the molecular weight (for cyanidin-3-glucoside, MW = 449 g mol^−1^). The limit of detection (2.9 mg/kg) was calculated from the refined wheat bread extracts used as matrix blanks.

#### 2.5.3. Total Phenolic and Total Flavonoid Content

Freeze-dried bread (2 g) was extracted using 20 mL of 80% methanol, vortexed for 5 min, sonicated at 40 kHz for 30 min, shaken at 200 rpm for 1 h, centrifuged at 4000 rpm for 15 min, and filtered through a 0.45 µm filter, following the protocol by Velioglu et al. [21] with some modifications. TPC was determined by the Folin–Ciocalteu method [8] and read at 760 nm against a gallic acid curve (0–50 µg/mL); the results are expressed as mg gallic acid equivalents per 100 g dry weight (mg GAE/100 g DW). TFC was determined by the AlCl_3_-NaNO_2_ method [22] and read at 502 nm against a quercetin curve; the results are expressed as mg quercetin equivalents per 100 g dry weight (mg QE/100 g DW).

#### 2.5.4. Antioxidant Activity

Extracts were prepared as in Section 2.5.3. Antioxidant capacity was assessed by ABTS and DPPH radical-scavenging assays. For ABTS, an ABTS·^+^ solution (7.4 mM ABTS, 2.6 mM K_2_S_2_O_8_) was generated in the dark for 12 h, diluted, combined with sample or buffer (7:1, *v*/*v*), held in the dark for 6 min, and read at 729 nm [23]. For DPPH, 100 µL of sample was added to 1.9 mL of 186 µmol/L DPPH, held at room temperature for 30 min, and read at 517 nm [24]. The radical-scavenging activity was calculated as follows:

(4) $$\mathrm{Scavenging}\;\mathrm{capacity}\left(\%\right)=\frac{\mathrm{Ac}-\mathrm{As}}{\mathrm{Ac}}\times 100$$

where Ac and As represent the absorbance of the radical plus buffer and the radical plus samples, respectively.

#### 2.5.5. In Vitro Functional Properties

The alcohol dehydrogenase (ADH) activation activity was determined as described by Uriho et al. [9], with some modifications. For both assays, extracts were prepared as in Section 2.5.3. Briefly, 1.5 mL of pyrophosphate buffer (0.1 M, pH 8.8), 0.1 mL of 0.25 U/mL ADH, 0.5 mL of ethanol (11.5%, *v*/*v*), and 0.1 mL of the sample were mixed at 25 °C, followed by the addition of 1.0 mL of 0.01 M NAD+ to initiate the reaction. Absorbance at 340 nm was measured using a microplate reader and again after 15 min. The percentage activation of ADH activity was calculated using Equation (5):

(5) $$\mathrm{ADH}\;\mathrm{activation}\;\mathrm{activity}\left(\%\right)=\frac{A1-A0}{A0}\times 100$$

In this equation, A1 indicates the absorbance of the tested sample while A0 stands for the absorbance of the control without the sample.

The acetylcholinesterase (AChE) inhibition assay was performed according to a previously reported method [25] with modifications. Briefly, 20 µL of bread extract at various concentrations was combined with 180 µL of Ellman’s reagent solution. This solution contained 318 µM DTNB and 955 µM ATChI in 0.1 M phosphate buffer at pH 7.5. Subsequently, 50 µL of AChE solution was added to the mixture. The progress of the reaction was monitored by measuring the change in absorbance at 412 nm over a 10 min period. Control experiments were conducted by substituting the extract with 20 µL of the solvent. Galantamine was used as a standard reference inhibitor for AChE. The percentage of inhibition of AChE activity was calculated using the following formula (Equation (6)):

(6) $$\mathrm{AChE}\;\mathrm{inhibition}\left(\%\right)=\frac{A0-A1}{A0}\times 100$$

In this equation, A0 stands for the absorbance of the control without the sample, while A1 indicates the absorbance of the tested sample.

### 2.6. Physical Properties of Bread

#### 2.6.1. Specific Volume and Crumb Structure

The specific volume of the bread was measured 2 h after baking. The weight of the bread loaves was first measured using a digital balance, and the volume of the bread samples was then determined by the rapeseed displacement method in accordance with AACC method 10-05.01. The specific volume (mL/g) of the bread was calculated as the ratio of loaf volume (mL) to loaf weight (g).

The method for bread crumb structure analysis was consistent with the study of Li et al. [26], with a few minor modifications. Images of bread crumb samples were captured using a printer scanner (HP LaserJet Pro 100 color MFP M175nw, Hewlett-Packard Company, Palo Alto, CA, USA), and the central 2 × 2 cm region of each image was cropped. The image was then binarized in ImageJ (Version 1.5.0_09) using the Otsu algorithm with an 8-bit grayscale threshold. The cell density (CD, cells/cm^2^) and mean cell area (MA, mm^2^) of the crumb were calculated from the number of pores per square centimeter of the image (cell/cm^2^), while the pore area fraction was defined as the ratio of the pore area to the total selected area (%).

#### 2.6.2. Crumb Firmness and Staling Rate

Cooled loaves were packed in 40 µm LDPE zip-lock bags and stored in the dark at 25 ± 1 °C and 55 ± 5% RH for 7 days. Firmness was measured at day 0 (2 h after baking) and day 7, as described by Jin et al. [27], using a texture analyzer (Texture Pro CT v1.4, Brookfield, Middleboro, MA, USA) with a 36 mm cylindrical probe in double-compression TPA mode (pre-test 3 mm/s, test 1 mm/s, post-test 5 mm/s, trigger 5 g; two central slices compressed to 50%). The staling rate was calculated using Equation (7).

(7) $$\mathrm{Staling}\;\mathrm{rate}=\frac{\mathrm{crumb}\;\mathrm{hardness}\;\mathrm{at}\;\mathrm{day}\;7-\mathrm{crumb}\;\mathrm{hardness}\;\mathrm{at}\;\mathrm{day}\;0}{7}$$

#### 2.6.3. Color Analysis

The crumb color of the breads was measured with a colorimeter (CR-400, Konica Minolta Co, Tokyo., Japan) in the CIELAB color space. The instrument was calibrated with a white reference plate, and lightness (L*), redness (a*) and yellowness (b*) were measured in triplicate on a homogeneous surface of the crumb. Chroma (C*), hue angle (h°) and the total color difference relative to the refined white wheat control (ΔE*) were calculated for each replicate using Equations (8)–(10):

(8) $$C*=\sqrt{{a*}^{2}+{b*}^{2}}$$

(9) $$h{}^{\circ}=\tan^{-1}\left(\frac{b*}{a*}\right)$$

(10) $$\Delta E*=\sqrt{{\left(L*-{L*}_{0}\right)}^{2}+{\left(a*-{a*}_{0}\right)}^{2}+{\left(b*-{b*}_{0}\right)}^{2}}$$

where L*, a* and b* are the values of each sample and L*_0_, a*_0_ and b*_0_ are the mean values of the control bread (WB). The hue angle was calculated with the two-argument arctangent (ATAN2 in Excel), which places h° in the correct quadrant; this is equivalent to adding 180° to arctan(b*/a*) when a* is negative. ΔE* values above about 5 are generally regarded as clearly perceptible to consumers.

### 2.7. Statistical Analysis

The results of three independent bread making batches for each assay are presented as mean values. Differences among the four breads were assessed by one-way ANOVA with Duncan’s test (*p* < 0.05). The effects of whole black wheat and co-culture fermented Cili, and their interaction, were assessed by two-way ANOVA (general linear model, fixed factors; Supplementary Table S2). All ANOVAs were performed with SPSS 16.0 (SPSS Inc., Chicago, IL, USA). Pearson correlations and PCA were calculated on the four formulation means with OriginPro 2024 (OriginLab Corporation, Northampton, MA, USA) and are considered exploratory.

## 3. Results and Discussion

### 3.1. Dough pH and Total Titratable Acidity

**Figure 1:**
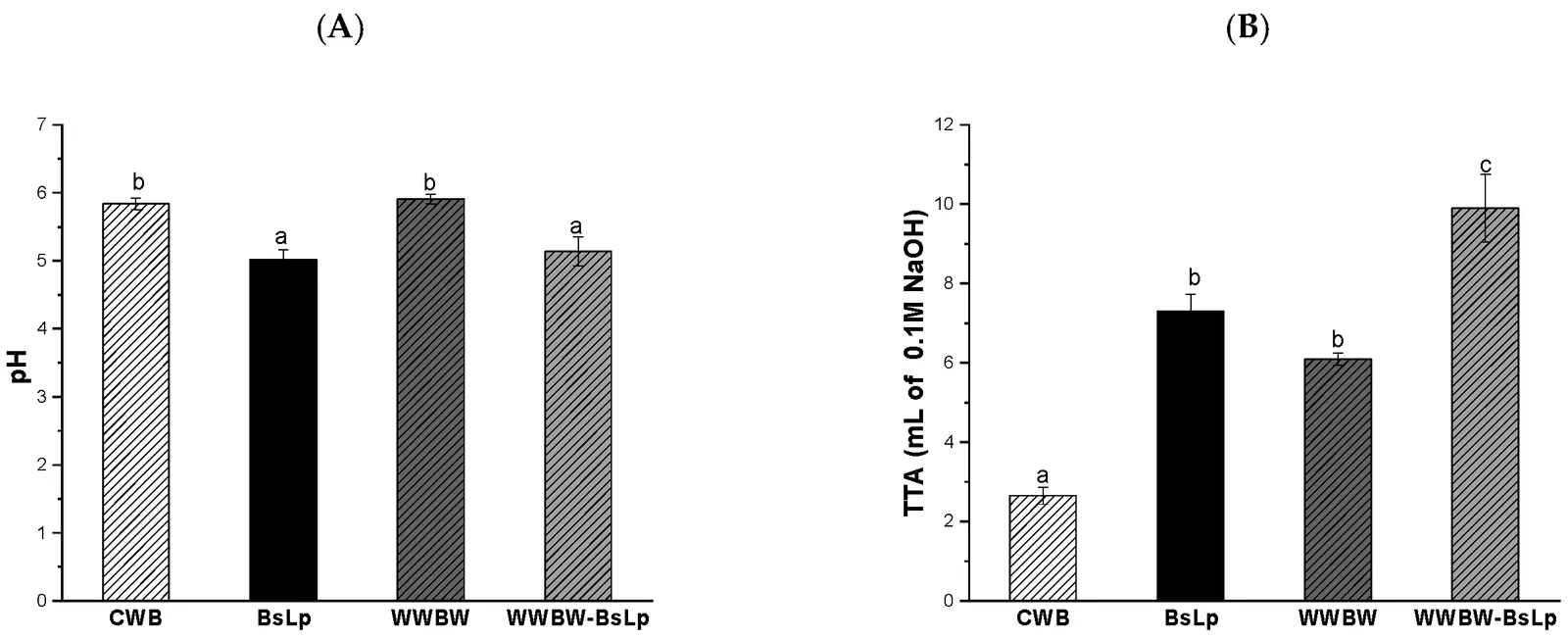
Effect of co-culture fermented Cili fruit on pH (**A**) and TTA (**B**) of white wheat–black wheat composite dough. The different small letters represented significant difference at *p* < 0.05.

Figure 1 presents the acidity of the unproofed doughs. Adding 30% co-fermented Cili markedly lowered dough pH, from 5.84 in the refined control (WB) to 5.02 in BsLp, and from 5.91 in the composite dough (WWBW) to 5.14 in WWBW-BsLp (*p* < 0.05). By contrast, replacing 50% of the refined flour with whole black wheat alone did not significantly affect pH; WB and WWBW showed no significant difference (Figure 1A). TTA showed a similar, though not identical, trend (Figure 1B). It increased from approximately 2.6 mL of 0.1 M NaOH in WB to 6.1 mL in WWBW and 7.3 mL in BsLp, with no significant difference between the latter two. TTA was highest in WWBW-BsLp at approximately 9.9 mL, nearly four times the control value (*p* < 0.05).

Because these measurements were taken before proofing, the differences reflect the ingredients’ acid load and buffering capacity, not yeast activity in the dough. The acidification of the Cili-containing doughs is attributable to organic acids carried over from the co-fermented Cili including lactic acid produced by *L. plantarum* during anaerobic fermentation [9], as well as the fruit’s intrinsic organic acids, including its exceptionally high ascorbic acid content [5,6]. By contrast, the black wheat doughs show that TTA and pH are not interchangeable: WWBW more than doubled the control’s TTA without lowering its pH. This is characteristic of bran-rich, wholemeal systems, where bran proteins, phytate, minerals, and phenolic acids provide titratable buffering groups. As a result, more alkali is needed to reach the titration end point, even when the free-acid concentration is low [2,28]. The same buffering explains why WWBW-BsLp, despite having the greatest total acid load (the highest TTA), did not reach a lower pH than BsLp: organic acids from the ferment were partly neutralized by the black wheat matrix.

The mildly acidic environment in the co-fermented doughs (pH 5.02–5.14) has several implications for the results that follow. It is close to the acidic optimum of the β-glucosidase and related hydrolases measured in Section 3.3, supporting their activity during proofing. Together with these enzymes, it also contributes to the weakening of the gluten–starch network observed in the rheological data (Section 3.2) [28]. In addition, the less alkaline conditions favor anthocyanin retention during baking [29]. Importantly, the pH of the co-fermented doughs remained above the strongly acidic range typical of traditional sourdoughs.

### 3.2. Dough Rheological Properties

**Figure 2:**
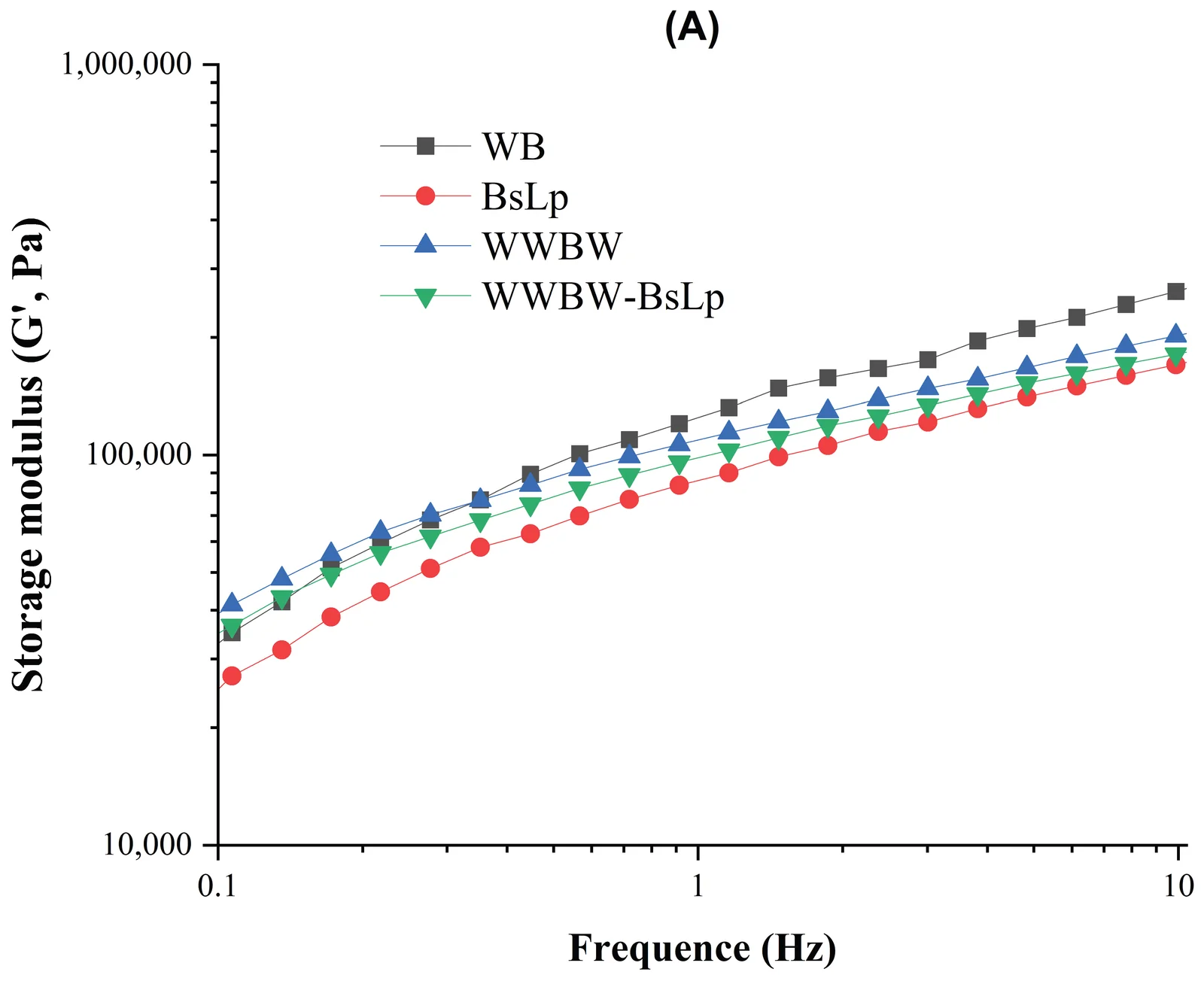

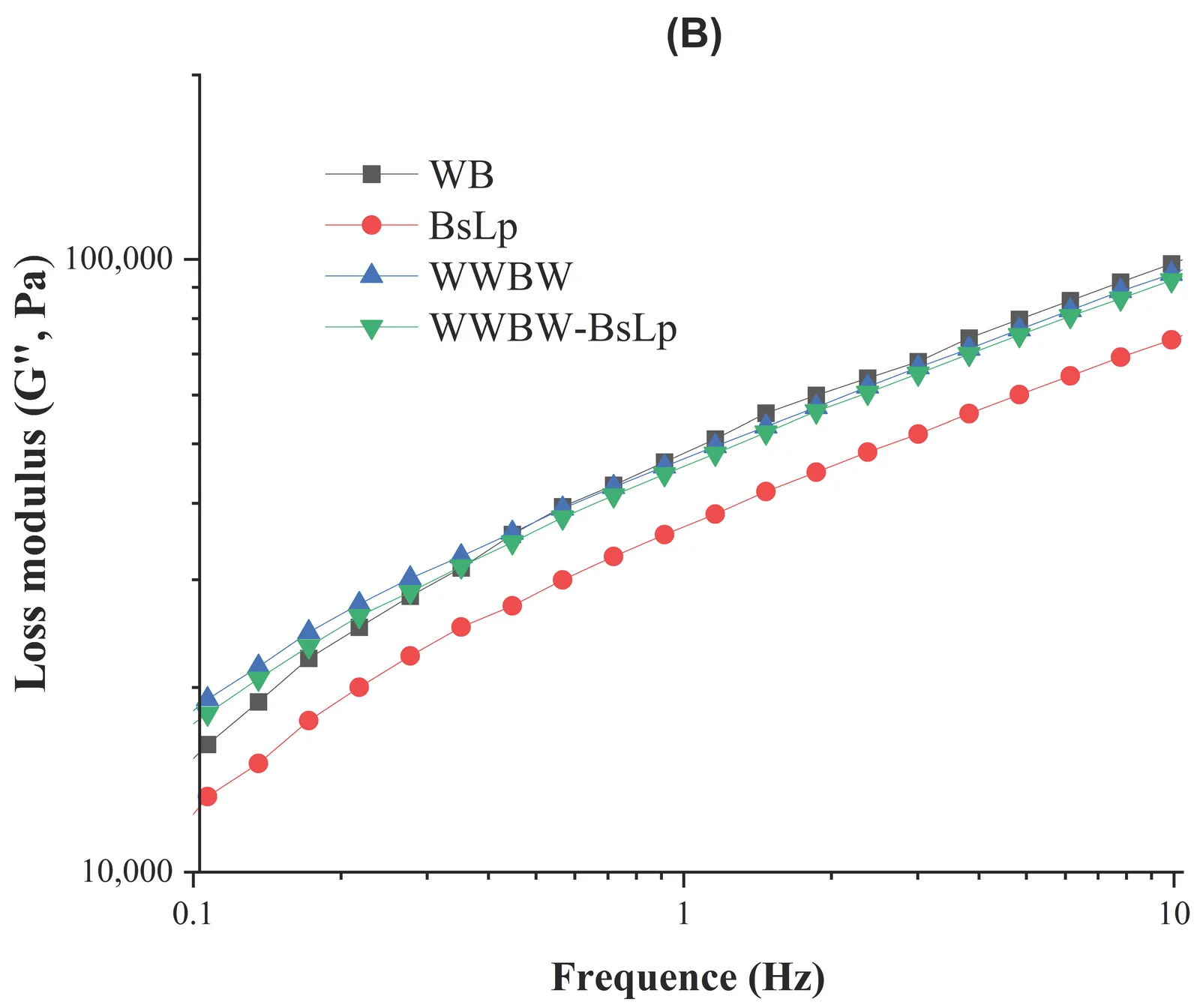

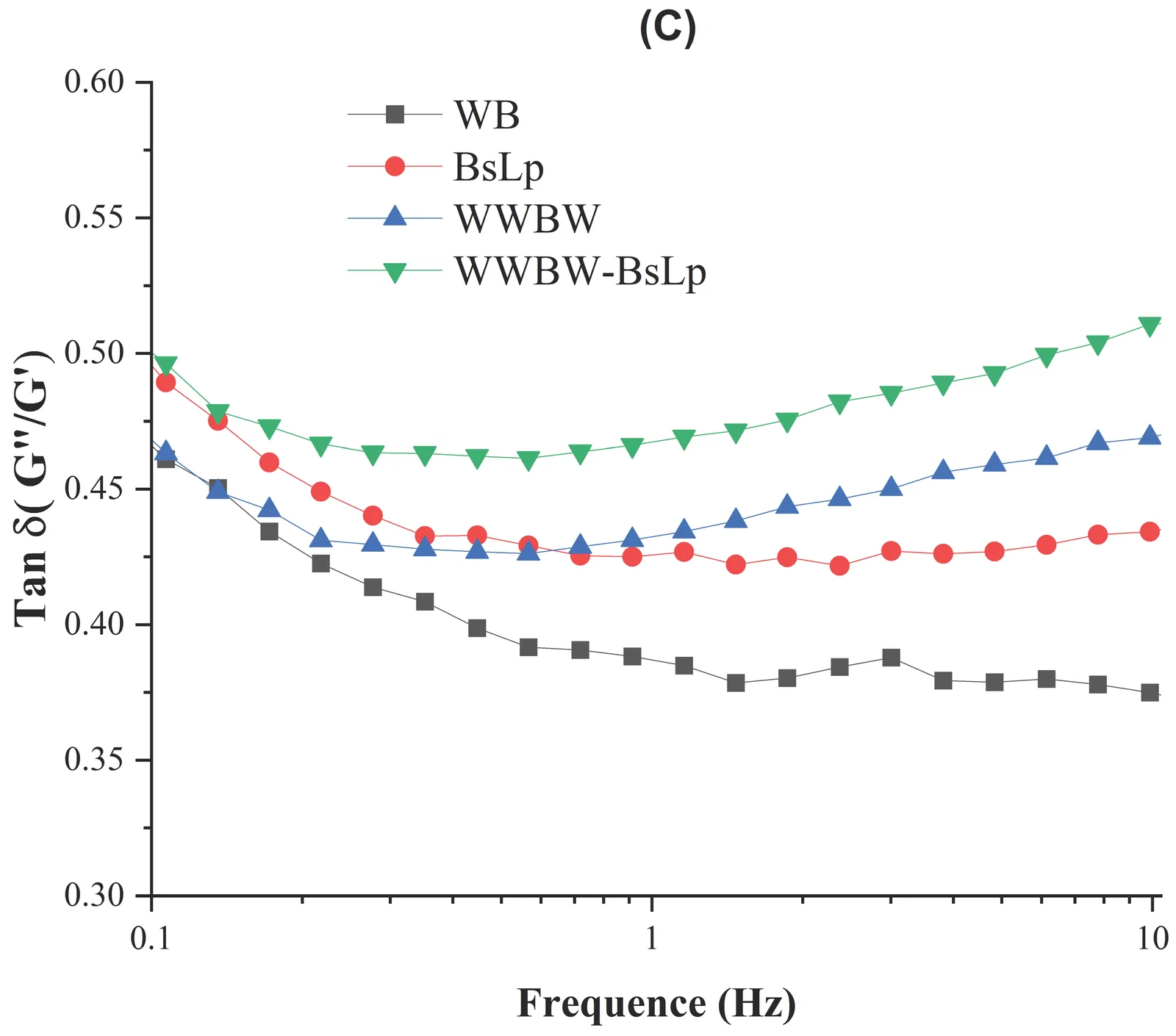
The changes in rheological properties, including storage modulus (G′) (**A**), loss modulus (G″) (**B**), and loss tangent (tan δ) (**C**) for different doughs. WB, refined wheat control dough; BsLp, refined wheat dough with 30% Cili fruit co-culture fermented by *B. subtilis* and *L. plantarum*; WWBW, refined wheat–whole black wheat composite dough; WWBW-BsLp, composite dough with 30% co-culture fermented Cili fruit.

The viscoelastic behavior of the four doughs (WB, BsLp, WWBW, WWBW-BsLp) was characterized by frequency sweeps from 0.1 to 10 Hz (Figure 2). Across all samples, the storage modulus (G′) exceeded the loss modulus (G″) over the entire frequency range, and both moduli increased with frequency, indicating that all doughs behaved as weak viscoelastic gels dominated by elastic, solid-like rather than viscous character (Figure 2A,B). The refined control WB exhibited the highest G′, consistent with a continuous, well-hydrated gluten network. Replacing 50% of the refined flour with whole black wheat (WWBW) lowered G′, in line with the dual action of bran-rich fractions described by Li et al. [2]: coarse bran particles and insoluble fiber physically interrupt the continuous gluten matrix and compete with gluten for water, so that network dilution outweighs any particle reinforcement at this substitution level. Consistent with these modulus changes, the loss tangent (tan δ = G″/G′) was lowest in the refined control and increased progressively in the composite and co-fermented doughs (Figure 2C), with the highest tan δ in WWBW-BsLp indicating the comparatively most viscous, least elastic network. An excessively weakened gluten network limits gas retention and loaf volume, as reflected in the bread quality data (Section 3.7).

Incorporating 30% co-fermented Cili (BsLp and WWBW-BsLp) further lowered G′ and increased tan δ. Several factors may explain this: (i) the lower dough pH (Section 3.1), which increases the net charge of gluten proteins and weakens protein–protein interactions; (ii) partial proteolysis and starch hydrolysis by hydrolases in the fermented ingredient; (iii) competition for water from Cili fiber and pectin; (iv) further dilution of gluten by non-gluten solids; and (v) the higher total water content of Cili-containing doughs (about 69.5% vs. 60.0% on a flour basis; Section 2.3), which alone would be expected to lower G′ and increase tan δ. The contributions of these factors cannot be separated in the present design. Organic-acid- and enzyme-rich sourdoughs have been reported to reduce dough elasticity [9,28].

### 3.3. Enzyme Activities in Dough

Three hydrolases central to matrix restructuring and bioactive release (β-glucosidase, feruloyl esterase and α-amylase) were quantified in the non-proofed and proofed doughs at 0 and 90 min (Table 2). During the 90 min proof, β-glucosidase activity increased significantly in all four doughs (e.g., from 3.91 to 4.41 U/g in WB and from 4.29 to 5.00 U/g in WWBW-BsLp). The α-amylase activity increased significantly in BsLp, WWBW and WWBW-BsLp but did not change significantly in WB (1.06 vs. 1.03 U/g; *p* > 0.05). Feruloyl esterase activity increased significantly only in WWBW-BsLp (0.15 to 0.17 U/g). Several factors may explain the increase in activity during proofing. First, β-glucosidase activity also increased in WB, which contained no fermented ingredient. The increase (+0.50 U/g) was similar to that observed in the other doughs (+0.70 to +0.77 U/g), suggesting that much of the rise came from sources other than the fermented ingredient. Endogenous cereal enzymes, concentrated in the bran and aleurone, as well as baker’s yeast enzymes, are likely contributors [30]. This is supported by the finding that WWBW, which contained no fermented Cili, reached a β-glucosidase activity after proofing (4.75 U/g) similar to that of BsLp (4.82 U/g). Second, gradual hydration of the dough during proofing may improve enzyme extractability, resulting in more activity being recovered at 90 min than immediately after mixing. Third, the lower pH of the doughs containing fermented Cili (5.02–5.14; Section 3.1) is closer to the reported pH optima of fungal and bacterial β-glucosidases and may therefore favor their activity. Finally, microbial enzymes introduced with the fermented ingredient may contribute to the activity measured in the dough [9].

After 90 min of proofing, differences among the formulations were modest. β-glucosidase activity in WWBW-BsLp was about 13% higher than in WB. α-amylase activity differed significantly from WB only in BsLp (1.14 vs. 1.03 U/g). Feruloyl esterase activity differed significantly from WB only in WWBW-BsLp (0.17 vs. 0.11 U/g). In addition, the assays measured potential activity under standardized conditions (pH, temperature, and synthetic substrates), not activity within the dough during proofing and baking. The higher activities are therefore consistent with the possibility that these hydrolases may contribute to increased release of bound phenolics, solubilization of cell-wall polysaccharides, and dough softening, as reported for cereal fermentations with *Bacillus* and lactic acid bacteria [7,8,18].

### 3.4. Changes in Dietary Fiber Composition in Dough and Bread

Total, insoluble, and soluble dietary fiber (TDF, IDF, SDF) were determined in both the doughs and the corresponding breads (Table 3). As expected, whole black wheat was the dominant fiber source: dough IDF rose from 2.19 g/100 g in the control to 4.66 g/100 g in WWBW and 6.77 g/100 g in WWBW-BsLp, and dough TDF increased correspondingly from 2.67 to 9.56 g/100 g, reflecting the bran and cell-wall polysaccharides of the pigmented grain [1]. The fermented Cili ingredient markedly increased dough SDF, from 0.48 to 2.77 g/100 g in refined wheat dough and from 0.70 to 2.79 g/100 g in composite dough. Since the increase was similar in both matrices, and the ingredient’s fiber composition before and after fermentation—as well as that of an unfermented Cili control—was not determined, the increase most likely reflects soluble fiber (e.g., pectic polysaccharides) contributed by the Cili fruit itself. The partial solubilization of cell-wall polysaccharides during fermentation, as reported for lactic acid bacteria and *Bacillus* in bran-rich substrates [18], remains possible but was not demonstrated here. Baking significantly increased IDF in all four formulations and significantly decreased SDF in WB, BsLp, and WWBW-BsLp, but not in WWBW (0.70 vs. 0.64 g/100 g DW; *p* > 0.05) (Table 3). TDF increased significantly after baking in WB, BsLp, and WWBW, but remained unchanged in WWBW-BsLp (9.56 vs. 9.46 g/100 g DW), suggesting that fiber in this bread shifted from the soluble to the insoluble fraction. Several processes may explain these changes: heat-induced aggregation of solubilized polysaccharides with proteins or polyphenols; formation of retrograded (resistant) starch, some of which AOAC 991.43 recovers as IDF; and formation of Maillard reaction products that assay enzymes cannot hydrolyze. AOAC 991.43 also fails to recover the low-molecular-weight soluble fiber that remains soluble in 78% ethanol, so small changes in SDF should be interpreted cautiously. Overall, WWBW-BsLp had the highest TDF (9.46 g/100 g DW) and SDF (2.29 g/100 g DW) among the four breads. The physiological effects of this fiber on glycemia or cholesterol were not assessed in this study.

### 3.5. Bioactive Composition of Breads

The bioactive composition, represented by the contents of GABA, TAC, TPC, and TFC in bread, is presented in Table 4.

GABA content. γ-Aminobutyric acid (GABA) was quantified to assess the functional enrichment achieved by adding co-culture fermented Cili fruit (Table 4). GABA content increased from 5.71 mg/kg DW in WB to 54.89 in WWBW, 299.50 in BsLp, and 312.19 mg/kg DW in WWBW-BsLp (all *p* < 0.05; Table 4). Thus, fermented Cili was the primary source of GABA, while black wheat contributed much less. A significant black wheat × Cili interaction was observed (*p* = 0.005): fermented Cili increased GABA by 294 mg/kg DW in refined wheat bread, compared with 257 mg/kg DW in the composite bread, indicating that the two ingredients did not act synergistically to increase GABA. The Cili substrate contained 270.94 ± 18.20 mg/kg GABA before fermentation and 528.10 ±16.84 mg/kg after co-culture fermentation [9]. In an earlier study, refined wheat bread contained 177.60 ± 8.98 mg/kg DW GABA with unfermented Cili and 327.84 ± 30.53 mg/kg DW GABA with co-fermented Cili [10]. Therefore, both the fruit’s intrinsic GABA and GABA produced during fermentation contribute to the bread’s GABA content. *L. plantarum* glutamate decarboxylase most likely produces the additional GABA, which is most active under mildly acidic conditions; *B. subtilis* proteolysis may also increase the supply of free glutamate [14,16]. However, this study did not measure GAD activity, proteolysis, or glutamate content in the bread. Moreover, for this bread, a 100 g serving of WWBW-BsLp could provide approximately 18.09 mg of GABA. In vivo studies are needed to establish its physiological relevance.

Total anthocyanin content (TAC). Anthocyanins were detected only in the black wheat breads (23.23 ± 1.25 mg/kg DW in WWBW and 25.30 ± 0.43 mg/kg DW in WWBW-BsLp). As expected, they were not detected in WB or BsLp (considered as blank), because anthocyanins are confined to the pericarp and aleurone of pigmented wheat [3,4]. TAC was slightly but significantly higher in WWBW-BsLp than in WWBW (*p* < 0.05), potentially due to either of two mechanisms. First, the lower dough pH resulting from the fermented ingredient may favor the more stable flavylium form of anthocyanins and reduce thermal degradation during baking [31,32]. Second, cell-wall-degrading enzymes may improve the extractability of pigments bound to the matrix. Since TAC was not measured in the ingredients or in the dough before baking, anthocyanin retention could not be calculated, and the present data cannot confirm either mechanism.

TPC and TFC. Total phenolic content (TPC) and total flavonoid content (TFC) were measured to quantify the phenolic pool available for antioxidant activity (Table 4). Both indices followed the order WWBW-BsLp > BsLp > WWBW > WB. TPC rose from 70.70 ± 9.07 mg GAE/100 g DW in the refined control to 197.78 ± 13.86 (WWBW), 283.29 ± 6.09 (BsLp), and 484.79 ± 11.69 mg GAE/100 g DW in WWBW-BsLp, while TFC increased in parallel from 16.99 ± 2.01 to 57.95 ± 2.86 mg QE/100 g DW. The increases in TPC and TFC mainly reflect the addition of two phenolic-rich ingredients: Cili fruit, one of the richest known fruit sources of phenolics and flavonoids [5], and whole black wheat bran [1,33]. In refined wheat bread, co-fermented Cili yielded only slightly higher TPC than unfermented Cili [10]. Two-way ANOVA showed a significant positive interaction between black wheat and Cili for TPC (*p* < 0.001) and TFC (*p* = 0.014): fermented Cili increased TPC by 213 mg GAE/100 g DW in refined wheat bread and by 287 mg GAE/100 g DW in composite bread. One possible explanation is that β-glucosidase and feruloyl esterase in the dough released bound phenolics from black wheat bran. However, free and bound phenolic fractions were not analyzed, so this mechanism remains to be confirmed. Future work characterizing individual compounds would clarify which compounds change.

### 3.6. In Vitro Antioxidant Activity and Functional Properties of Breads

**Figure 3:**
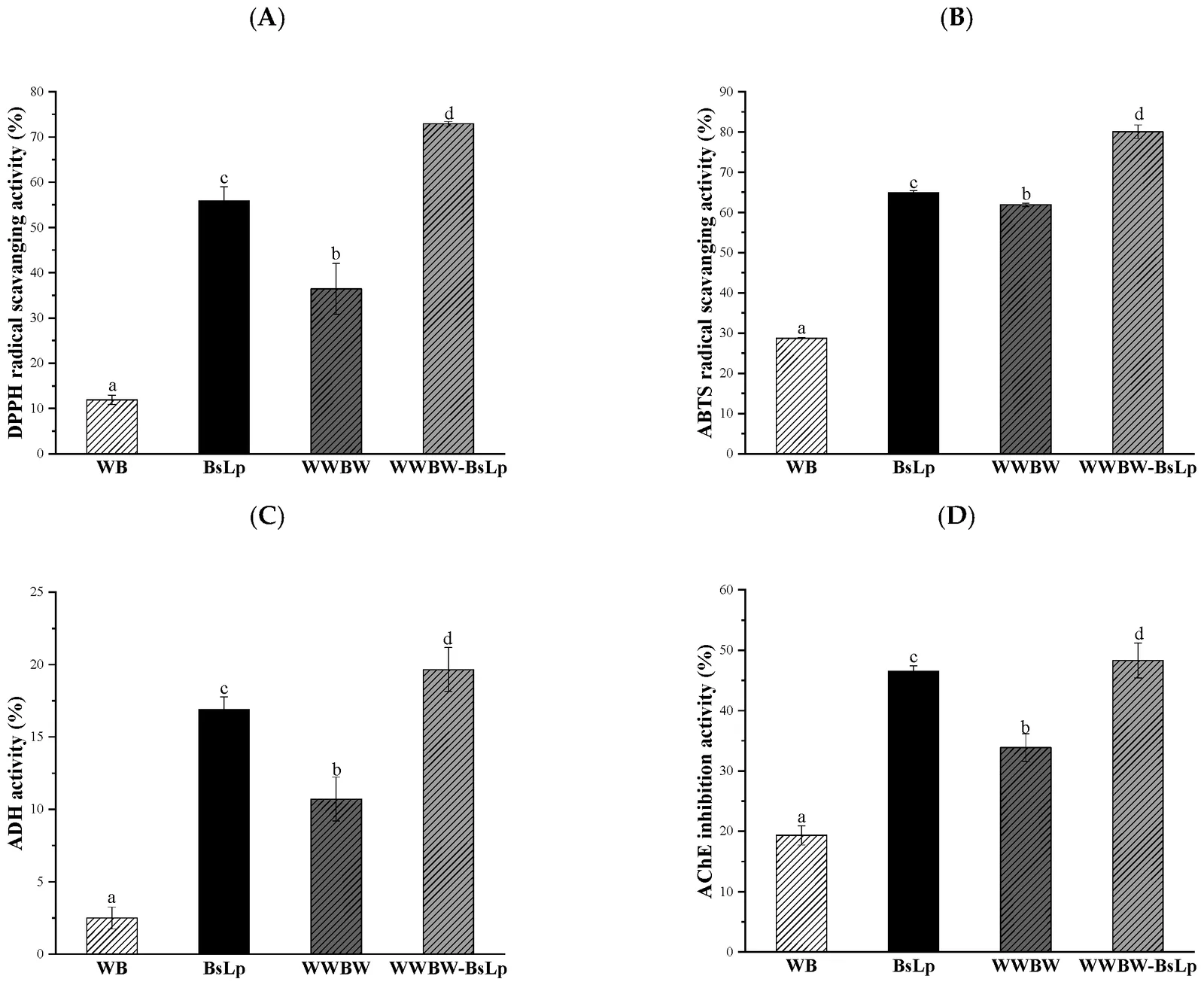
DPPH (**A**) and ABTS (**B**) radical-scavenging activity, ADH activation (**C**), and AChE inhibition (**D**) of breads enriched with whole black wheat and/or co-culture fermented Cili fruit. Mean ± SD (n = 3). Different superscript letters within a column indicate significant differences (*p* < 0.05). WB, refined wheat control bread; BsLp, refined wheat bread with 30% Cili fruit co-culture fermented by *B. subtilis* and *L. plantarum*; WWBW, refined wheat–whole black wheat composite bread; WWBW-BsLp, composite bread with 30% co-culture fermented Cili fruit.

The antioxidant capacity of the breads was assessed using the ABTS·^+^ and DPPH radical-scavenging assays (Figure 3B and Figure 3A, respectively). Both assays ranked the breads identically: WWBW-BsLp > BsLp > WWBW > WB (*p* < 0.05). DPPH scavenging rose from 11.93 ± 1.05% in WB to 36.43 ± 5.62% in WWBW, 55.89 ± 3.11% in BsLp, and 72.92 ± 0.49% in WWBW-BsLp. ABTS scavenging showed the same ranking, increasing from 28.75 ± 0.13% in WB to 80.04 ± 1.67% in WWBW-BsLp. Fermented Cili had a greater effect than black wheat: it increased DPPH scavenging by 44 percentage points in refined wheat bread and 36 points in composite bread, compared with a 17–25-point increase from black wheat. The interaction between black wheat and fermented Cili was not significant (two-way ANOVA, *p* = 0.08) (Table S2), indicating that the ingredients acted approximately additively in their effects on DPPH scavenging. This ranking matches that of TPC and TFC (Table 4), consistent with reports that phenolic compounds and flavonoids contribute to radical scavenging in pigmented cereals and fermented plant products [7,14]. Anthocyanins alone cannot explain the ranking: although WWBW contained anthocyanins, it scavenged less than BsLp, which contained none (Table 4). The release of free phenolics from glycosylated or ester-bound forms by β-glucosidase and feruloyl esterase may also contribute [7,8]. However, because we did not analyze free and bound phenolic fractions, this remains a hypothesis. Percentage scavenging also depends on extract concentration and assay conditions, and it approaches a plateau at high activity, compressing differences among the most active samples. Therefore, compare these values only within this study, not with absolute values reported elsewhere. Section 3.8 explores the relationships between antioxidant activity and bioactive composition.

Two in vitro functional endpoints associated with the bioactive fractions, alcohol dehydrogenase (ADH) activation and acetylcholinesterase (AChE) inhibition, were measured to assess potential physiological relevance beyond antioxidant capacity (Figure 3C,D). Both increased monotonically across the series (WB ˂ WWBW ˂ BsLp ˂ WWBW-BsLp) and differed significantly at each step (*p* < 0.05): ADH activation rose from 2.5 ± 0.75% (WB) to 10.71 ± 1.51% (WWBW), 16.87 ± 0.88% (BsLp), and 19.64 ± 1.51% (WWBW-BsLp), and AChE inhibition from 19.32 ± 1.59% to 33.86 ± 2.25%, 46.50 ± 0.94%, and 48.30 ± 2.90%, respectively.

The ADH and AChE results show the same ranking as TPC, TFC, and GABA. Because these are cell-free enzyme assays, they indicate that the bread extracts can modulate purified enzyme activity under the assay conditions; they do not establish physiological, hepatoprotective, or neurological effects. Phenolic compounds and flavonoids from Rosa roxburghii have previously been linked to ADH activation and AChE inhibition in vitro [5,13,31,32]. However, this study did not assess the activities of individual compounds or fractions. Therefore, the responsible constituents and whether these effects persist after digestion and absorption require investigation in cell and in vivo models.

### 3.7. Bread Physical Quality

**Figure 4:**
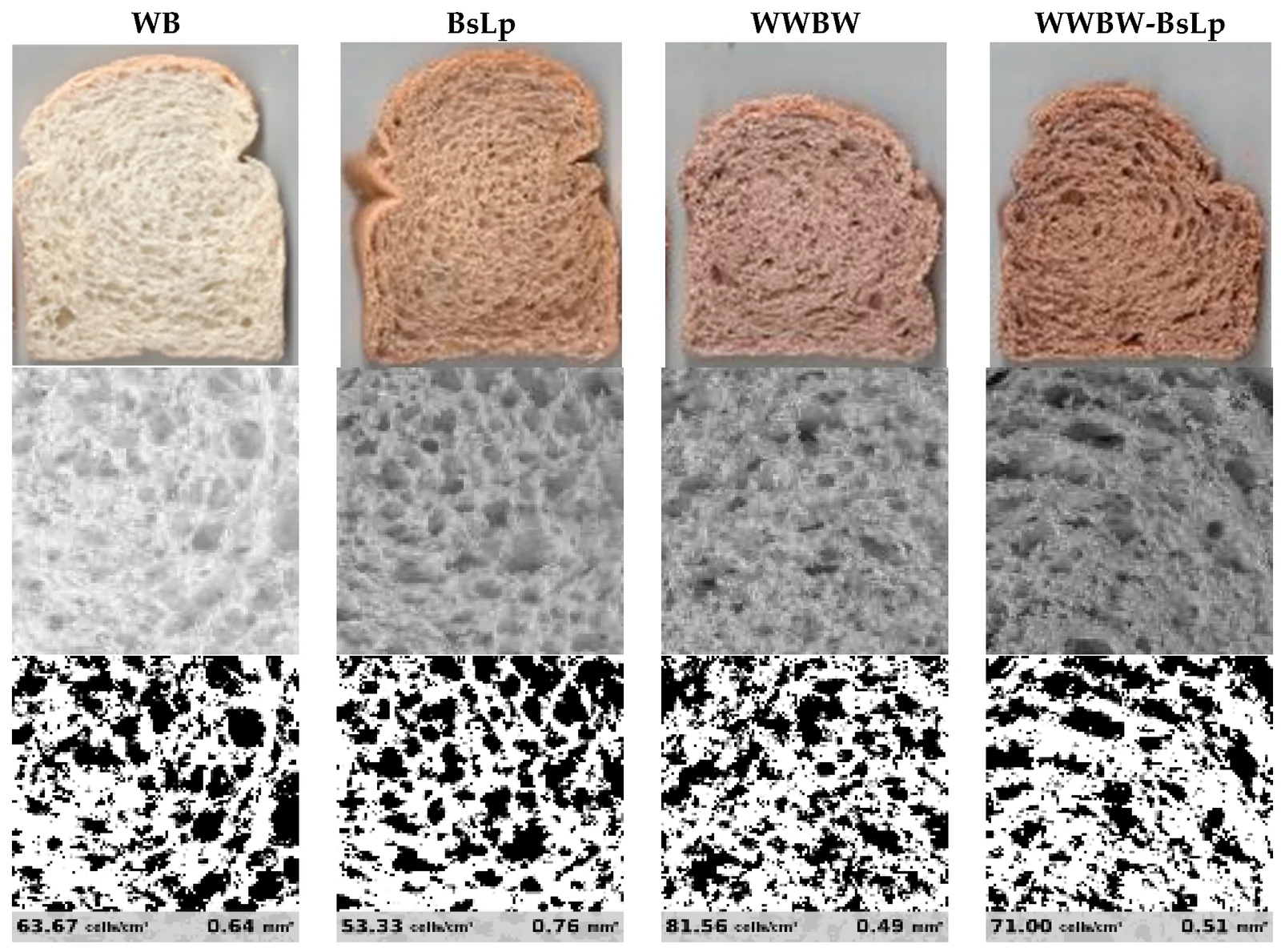
Binary-converted images of bread crumb cross-sections representing the gas cell structures of bread samples. The number in the lower-left corner of each image is the cell density (CD, in cells/cm^2^), whereas the number in the lower-right corner is the mean cell area (MA, in mm^2^). Both indices were averaged across a batch of breads in triplicate. WB, refined wheat control bread; BsLp, refined wheat bread with 30% Cili fruit co-culture fermented by *B. subtilis* and *L. plantarum*; WWBW, refined wheat–whole black wheat composite bread; WWBW-BsLp, composite bread with 30% co-culture fermented Cili fruit.

The physical properties of the composite breads containing co-culture fermented Cili fruits were determined, and the results are shown in Table 5 and Figure 4.

#### 3.7.1. Specific Volume, Texture and Anti-Staling Properties

Specific volume is an important measure of bread quality and provides a quantitative indication of baking performance [34]. The inclusion of whole black wheat reduced loaf specific volume from 5.13 mL/g in the refined control to 3.94 mL/g (WWBW), a decline of roughly 23% (Table 5), characteristic of bran-rich formulations, in which gluten dilution and physical disruption of the protein network by bran particles impair gas retention [2]. Adding co-culture fermented Cili partially mitigated this reduction: WWBW-BsLp increased to 4.17 mL/g, and in the refined wheat base, BsLp (5.11 mL/g) was statistically comparable to the control. This aligns with the loosening of the gluten–starch matrix and the supply of fermentable sugars via amylase, as discussed in Section 3.1 and Section 3.2. A similar pattern was observed for texture: black wheat sharply increased day-0 crumb firmness (from 296 g in the control to 493 g in WWBW), whereas co-culture fermented Cili fruit lowered composite bread firmness to 410 g. Since the Cili-containing doughs had a higher total water content (Section 2.3), potentially resulting in a higher dough hydration, they could have generally given a softer crumb. Most strikingly, both co-culture fermented Cili fruit and black wheat substitution reduced the staling rate from 230.6 g/day in the control to 146–161 g/day in the substituted breads, indicating markedly slower crumb firming over the seven-day storage period. Also, the staling rate was also lower in WWBW, which contained no fermented ingredient, and did not differ significantly among BsLp, WWBW, and WWBW-BsLp, this effect cannot be attributed specifically to co-fermentation. In the Cili-containing breads, organic acids carried over with the ingredient, which can retard amylopectin retrogradation, the principal molecular cause of bread firming [28,35], together with the soluble fiber of these formulations, may have contributed.

#### 3.7.2. Crumb Color Features and Implication on Consumer Acceptance

The L*, a*, and b* parameters of the bread crumb color are shown in Table 5. Crumb color changed as expected with pigment and acid load: L* (lightness) fell from 75.1 in the control to 52.5 in WWBW-BsLp, while a* (redness) rose from −3.12 to +4.19, reflecting the anthocyanin pigmentation of black wheat together with Maillard and organic-acid effects; the elevated b* of BsLp is consistent with the yellow-orange pigment load of Cili [36]. All three modified breads differed clearly from the control (ΔE* = 12.2–23.6), well above about 5, the threshold above which consumers readily perceive color differences. Adding the fermented ingredient further changed the color of the composite bread by ΔE* = 8.9 (WWBW-BsLp vs. WWBW). Hue angle decreased from 108.7° in WB to 65.4° in WWBW-BsLp, shifting from greenish-yellow toward red-orange and reflecting the black wheat anthocyanins. The chroma of BsLp (19.2) was almost double that of the other breads (9.7–10.1), consistent with the yellow-orange pigments of Cili. Overall, adding co-fermented Cili fruit lowered the crumb firmness of the composite bread without significantly changing its specific volume or staling rate. However, this study did not assess the sensory properties of the bread. In a previous study, white wheat bread containing 30% co-fermented Cili (BsLp) scored highly on the overall acceptability, with a score close to that of the refined white wheat control [37]. This suggests that co-fermented Cili can be incorporated into refined wheat bread without impairing acceptability with a score close to that of the refined white wheat control [37]. Whether this also applies to the darker, firmer composite bread, where the flavors of black wheat and Cili combine, remains to be tested with both a trained panel and consumers in future studies.

#### 3.7.3. Bread Crumb Grain Features

Figure 4 shows images of the porous structure of bread crumb slices; the cell density (CD) and mean cell area (MA) are indicated below each image. The composite breads showed higher cell densities (WWBW 81.56 and WWBW-BsLp 71.00 cells/cm^2^) but smaller mean cell areas (0.49 and 0.51 mm^2^). By contrast, the white flour breads formed fewer but larger, better-expanded cells (WB 63.67 cells/cm^2^, 0.64 mm^2^; BsLp 53.33 cells/cm^2^, 0.76 mm^2^). The observations were consistent with the specific volume and texture data. Bran fragments appear to interrupt bubble coalescence and expansion, producing many small cells rather than a few large ones—the structural signature underlying the lower specific volume and higher firmness of the black wheat breads (Table 5). This pattern closely mirrors that reported by Packkia-Doss et al. [38], who attributed the volume loss in bran-supplemented breads to bran-induced changes in the crumb pore-size distribution. Collectively, these observations confirm that the differences captured by CD and MA are not incidental but reflect the direct morphological expression of reduced gas cell stability and expansion imposed by bran fragments.

### 3.8. Correlation and Principal Component Analysis

**Figure 5:**
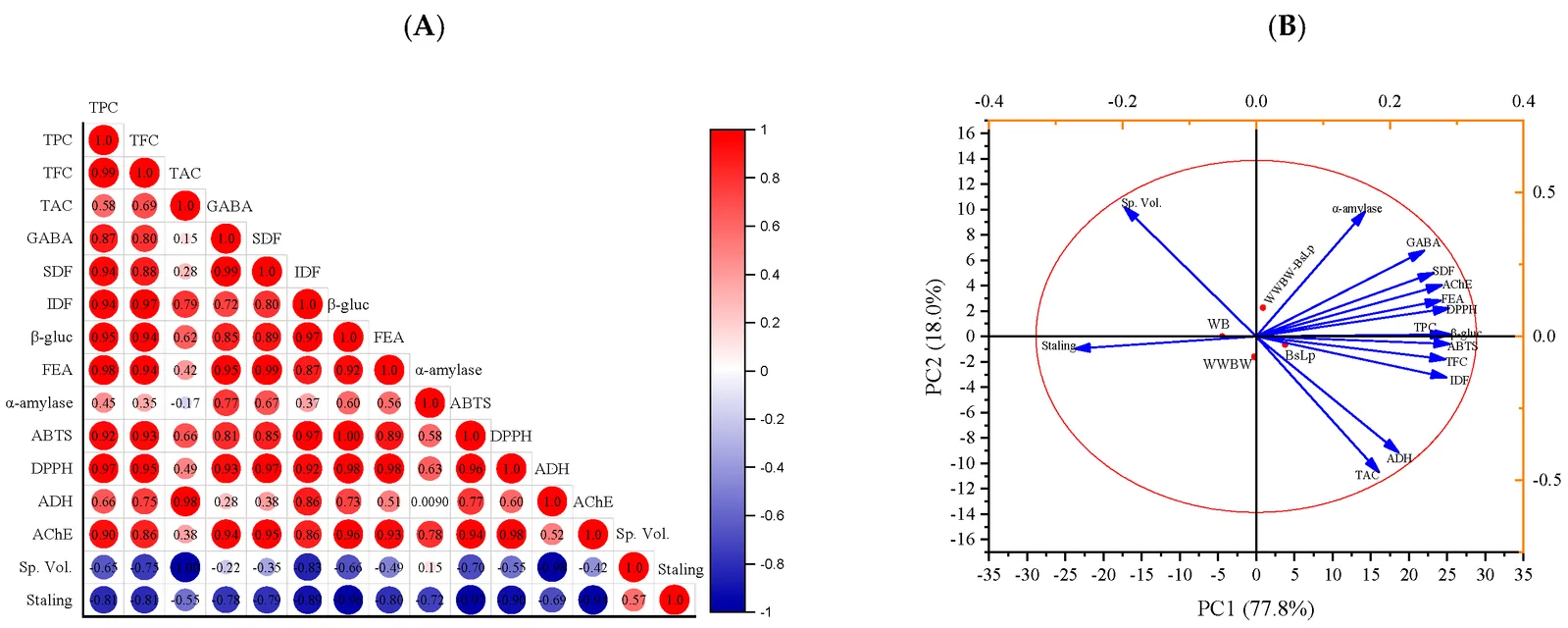
The Pearson correlation analysis (**A**) and PCA biplot (**B**) of co-variation among enzyme activity, bioactive, functional, and quality traits. The PCA biplot provides a two-dimensional overview of the data.

Pearson correlation and PCA were used to examine co-variation among the measured variables across the four formulations (Figure 5). Because the analyses used four formulation means, correlations were significant at *p* < 0.05 only when |r| ≥ 0.950. The results therefore describe patterns of co-variation but cannot establish causal or mechanistic relationships. Feruloyl esterase activity co-varied with TPC (r = 0.98), SDF (r = 0.99), TFC (r = 0.94), and GABA (r = 0.95); TPC, TFC, and GABA also co-varied with DPPH scavenging (r = 0.93–0.97). These associations are consistent with—but do not demonstrate—a role for dough hydrolases in phenolic release. Since the fermented Cili ingredient introduced enzymes, phenolics, GABA, and soluble fiber simultaneously, these variables would be expected to co-vary even in the absence of a causal link. TAC correlated only weakly with antioxidant activity (r = 0.49), as anthocyanins were not detected in the two refined wheat breads. Since TAC is absent in refined white wheat breads, it varies independently of the phenolics contributed by the fermented Cili ingredient and cannot alone explain the ranking among all four breads. Instead, TAC is best regarded as a marker for black wheat but not as a primary antioxidant contributor in this context.

Furthermore, the first two principal components explained 95.8% of the total variance (PC1, 77.8%; PC2, 18.0%). Because the analysis is based on four formulations and many variables, it is exploratory. PC1 separated the breads in the order WB < WWBW < BsLp < WWBW-BsLp and was associated with enzyme activities, TPC, TFC, SDF, GABA, and the antioxidant and functional assays. PC2 was dominated by TAC (loading ≈ 0.81) and separated the black wheat breads from the refined wheat breads. The PCA thus indicates that the fermented Cili ingredient and black wheat influenced different variable sets.

Limitations. The present design compared breads with and without co-fermented Cili and did not include breads containing unfermented Cili. Because Cili fruit is itself rich in phenolics, flavonoids, ascorbic acid, soluble polysaccharides and GABA, the differences reported here reflect the combined effect of adding the fruit and of its co-culture fermentation. The results from our companion study with refined wheat bread suggest that fermentation mainly increased GABA and soluble fiber, whereas TPC and TFC came mainly from the fruit [10]. These contributions should be confirmed in the composite system.

## 4. Conclusions

This study compared refined wheat breads with breads made from a 50:50 white-to-whole black wheat blend, prepared with or without 30% (flour basis) Cili fruit puree co-fermented with *Bacillus subtilis* and *Lactiplantibacillus plantarum*. In the composite bread, the fermented ingredient increased GABA (312.2 vs. 54.9 mg/kg DW), total phenolics (484.8 vs. 197.8 mg GAE/100 g DW), total flavonoids (58.0 vs. 32.3 mg QE/100 g DW), soluble dietary fiber (2.29 vs. 0.64 g/100 g DW), and DPPH radical-scavenging activity (72.9 vs. 36.4%). It also reduced crumb firmness (410 vs. 493 g) without significantly affecting specific volume (4.17 vs. 3.94 mL/g). Whole black wheat contributed anthocyanins and insoluble fiber but lowered specific volume by about 23%. Doughs containing the fermented ingredient had lower pH and higher β-glucosidase, feruloyl esterase, and α-amylase activities. These differences are consistent with, but do not demonstrate, enzymatic release of bound phenolics or softening of the dough matrix. Because breads containing unfermented Cili were not included, the reported effects reflect the combined contributions of the fruit and its fermentation. ADH activation and AChE inhibition were measured in cell-free assays and do not establish health effects. Future work should include unfermented Cili controls, standardize the total hydration for the dough, characterize the ingredient before and after fermentation, evaluate sensory properties and consumer acceptance (particularly sourness, astringency, color, and texture), and assess the bioavailability and in vivo effects of GABA and phenolic compounds.

## Figures and Tables

**Table 1:** Bread formulations.

	Refined White Wheat Flour (g)	Whole Black Wheat Flour (g)	Co-Culture Fermented Cili Fruit-Based Sourdough (g)	Yeast (g)	Water (g) *	Sugar (g)	Salt (g)	Butter (g)
WB	300	0	0	4.5	180	24	3	24
BsLp	300	0	90	4.5	135	24	3	24
WWBW	150	150	0	4.5	180	24	3	24
WWBW-BsLp	150	150	90	4.5	135	24	3	24

* Total hydration was not standardized. WB, refined white wheat control bread; BsLp, refined white wheat bread with 30% co-culture fermented Cili fruit-based sourdough; WWBW, refined white wheat–whole black wheat composite bread; WWBW-BsLp, composite bread with 30% co-culture fermented Cili fruit.

**Table 2:** Effect of co-fermented Cili fruit on dough enzyme activities (U/g) at 0 and 90 min of proofing.

	Enzyme Activity (U/g)					
	α-Amylase		β-Glucosidase		Feruloyl Esterase Activity	
Sample ID	0 min	90 min	0 min	90 min	0 min	90 min
WB	1.06 ± 0.02 ^bA^	1.03 ± 0.05 ^aA^	3.91 ± 0.04 ^aA^	4.41 ± 0.06 ^aB^	0.10 ± 0.01 ^aA^	0.11 ± 0.01 ^aA^
BsLp	1.07 ± 0.01 ^bA^	1.14 ± 0.02 ^bB^	4.05 ± 0.03 ^abA^	4.82 ± 0.05 ^bB^	0.13 ± 0.02 ^aA^	0.15 ± 0.02 ^abA^
WWBW	0.95 ± 0.01 ^aA^	1.06 ± 0.01 ^aB^	4.05 ± 0.14 ^aA^	4.75 ± 0.03 ^bB^	0.11 ± 0.01 ^aA^	0.12 ± 0.02 ^abA^
WWBW-BsLp	1.00 ± 0.02 ^aA^	1.07 ± 0.01 ^aB^	4.29 ± 0.05 ^bA^	5.00 ± 0.06 ^cB^	0.15 ± 0.04 ^aA^	0.17 ± 0.02 ^bB^

Mean ± SD (n = 3). Different lowercase letters within a column denote significant differences (*p* < 0.05); different uppercase letters denote significant differences (*p* < 0.05) between proofing times for the same parameter. WB, refined wheat control dough; BsLp, refined wheat dough with 30% Cili fruit co-culture fermented by *B. subtilis* and *L. plantarum*; WWBW, refined wheat–whole black wheat composite dough; WWBW-BsLp, composite dough with 30% co-culture fermented Cili fruit.

**Table 3:** Dietary fiber composition of dough and bread.

	Dietary Fiber Composition (g/100 g DW)					
	IDF		SDF		TDF	
Sample ID	Dough	Bread	Dough	Bread	Dough	Bread
WB	2.19 ± 0.05 ^aA^	2.70 ± 0.04 ^aB^	0.48 ± 0.05 ^aB^	0.30 ± 0.05 ^aA^	2.67 ± 0.11 ^aA^	3.01± 0.09 ^aB^
BsLp	2.99 ± 0.06 ^bA^	4.89 ± 0.04 ^bB^	2.77 ± 0.06 ^cB^	1.86 ± 0.06 ^cA^	5.77 ± 0.12 ^cA^	6.76 ± 0.11 ^cB^
WWBW	4.66 ± 0.05 ^cA^	5.31 ± 0.06 ^cB^	0.70 ± 0.06 ^bA^	0.64 ± 0.05 ^bA^	5.36± 0.12 ^bA^	5.95 ± 0.12 ^bB^
WWBW-BsLp	6.77 ± 0.04 ^dA^	7.17 ± 0.04 ^dB^	2.79 ± 0.04 ^cB^	2.29 ± 0.04 ^dA^	9.56 ± 0.08 ^dA^	9.46 ± 0.08 ^dA^

Mean ± SD (n = 3). Different lowercase letters within a column denote significant differences (*p* < 0.05); different uppercase letters denote significant differences (*p* < 0.05) for a specific parameter across different processing. WB, refined wheat control dough/bread; BsLp, refined wheat dough/bread with 30% Cili fruit co-culture fermented by *B. subtilis* and *L. plantarum*; WWBW, refined wheat–whole black wheat composite dough/bread; WWBW-BsLp, composite dough/bread with 30% co-culture fermented Cili fruit.

**Table 4:** Bioactive composition of control white bread and breads enriched with whole black wheat and/or co-culture fermented Cili (*Rosa roxburghii*) fruit.

	Bioactive Components of Bread			
Sample ID	GABA (mg/kg DW)	TAC (mg/kg DW)	TPC (mg GAE/100 g DW)	TFC (mg QE/100 g DW)
WB	5.71 ± 0.35 ^a^	n.d.	70.70 ± 9.07 ^a^	16.99 ± 2.01 ^a^
BsLp	299.50 ± 10.60 ^c^	n.d.	283.29 ± 6.09 ^c^	34.73 ± 2.31 ^b^
WWBW	54.89 ± 5.11 ^b^	23.23 ± 1.25 ^a^	197.78 ± 13.86 ^b^	32.32 ± 1.31 ^b^
WWBW-BsLp	312.19 ± 11.82 ^d^	25.30 ± 0.43 ^b^	484.79 ± 11.69 ^d^	57.95 ± 2.86 ^c^

Mean ± SD (n = 3). Different lowercase letters within a column denote significant differences (*p* < 0.05). n.d. (not detected). WB, refined wheat control bread; BsLp, refined wheat bread with 30% Cili fruit co-culture fermented by *B. subtilis* and *L. plantarum*; WWBW, refined wheat–whole black wheat composite bread; WWBW-BsLp, composite bread with 30% co-culture fermented Cili fruit.

**Table 5:** The specific volume, crumb firmness, staling rate, and crumb color of the breads.

		WB	BsLp	WWBW	WWBW-BsLp
Specific volume (mL/g)		5.13± 0.21 ^b^	5.11± 0.10 ^b^	3.94± 0.11 ^a^	4.17± 0.07 ^a^
Crumb firmness (g)		296.00 ± 33 ^a^	359.33 ± 7.50 ^ab^	492.66 ± 46.82 ^c^	409.66 ± 50.93 ^b^
Staling rate (g/day)		230.62 ± 8.39 ^b^	151.19 ± 4.34 ^a^	161.24 ± 13.35 ^a^	146.14 ± 5.28 ^a^
Crumb color	L*	75.10 ± 2.11 ^d^	60.68 ± 3.60 ^b^	61.18 ± 0.27 ^c^	52.53 ± 1.60 ^a^
	a*	−3.12 ± 0.09 ^a^	0.68 ± 0.21 ^b^	3.08 ± 0.42 ^c^	4.19 ± 0.11 ^d^
	b*	9.20 ± 0.52 ^a^	19.16 ± 0.31 ^b^	9.52 ± 0.71 ^a^	9.18 ± 0.46 ^a^
	C*	9.72 ± 0.59 ^a^	19.17 ± 0.32 ^b^	10.01 ± 0.80 ^a^	10.09 ± 0.45 ^a^
	hᵒ	108.73 ± 0.56 ^d^	87.96 ± 0.61 ^c^	72.13 ± 1.15 ^b^	65.43 ± 0.86 ^a^
	ΔE* vs WB	-	12.22 ± 1.04 ^a^	14.99 ± 1.47 ^b^	23.58 ± 1.04 ^c^

Mean ± SD (n = 3). Different lowercase letters within a row denote significant differences (*p* < 0.05). WB, refined wheat control bread; BsLp, refined wheat bread with 30% Cili fruit co-culture fermented by *B. subtilis* and *L. plantarum*; WWBW, refined wheat–whole black wheat composite bread; WWBW-BsLp, composite bread with 30% co-culture fermented Cili fruit.

## Data Availability

The data presented in this study are available on request from the corresponding author. The data are not publicly available due to privacy restrictions.

## Supplementary Materials

The following supporting information can be downloaded at https://www.mdpi.com/article/10.3390/foods15203705/s1, Table S1: The pH, moisture content, titratable acidity, GABA concentration, microbial counts, and selected enzyme activities in the fermented Cili used as an ingredient in bread making (Table 1); Table S2: Two-way ANOVA of the effects of whole black wheat, co-culture fermented Cili fruit and their interaction on the dietary fiber, bioactive composition, antioxidant and in vitro functional activities, and physical quality of the breads.
