# Supplementary material for: Cili (Rosa roxburghii Tratt.) Fruit and Its Co-Culture Fermentation with Bacillus subtilis and Lactiplantibacillus plantarum Improves the Nutritional, Functional and Bioactive Quality of White–Black Wheat Composite Bread
Source: Foods. 2026 Oct 17;15(20):3705. doi: 10.3390/foods15203705 (PMC13704317; doi:10.3390/foods15203705)
Supplement: Supplementary file 1 [file foods-15-03705-s001.zip › foods-4584421-supplementary.pdf]

Supplementary materials

Table S1. The pH, moisture content, titratable acidity, GABA concentration, microbial counts, and selected enzyme activities in the fermented Cili used as an ingredient in bread making (Table 1).

**Fermented Cili**	**Value**
pH	4.86 ± 0.02
Titratable acidity (mL)	19.30 ± 0.99
Moisture content (%)	81.68 ± 0.15
GABA content (mg/Kg FW)	111.65 ± 9.19
Microbial count (Log CFU/mL)	10.83 ± 0.08
**Enzyme activities**	
Esterase enzyme (U/mL)	0.80 ±0.02
β-glucosidase enzyme (U/mL)	28.76 ± 1.36
GAD activity (U/g F.W)	1.83 ± 0.00373

Table S2. Two-way ANOVA of the effects of whole black wheat, co-culture fermented Cili fruit and their interaction on the dietary fiber, bioactive composition, antioxidant and in vitro functional activities, and physical quality of the breads.

**Parameter**	**Black wheat**	**Fermented Cili**	**Black wheat × fermented Cili**	**Interaction contrast**[b]	
	F (p)	*F (p)*	*F (p)*		
Dietary fiber					
IDF (g/100 g DW)	14585 (< 0.001)	10081 (< 0.001)	64.4 (< 0.001)	−0.32	Significant
SDF (g/100 g DW)	371 (< 0.001)	5372 (< 0.001)	16.2 (0.004)	+0.18	Significant
TDF (g/100 g DW)	4565 (< 0.001)	7496 (< 0.001)	3.0 (0.122)	−0.15	Not significant
GABA (mg/kg DW)	87.6 (< 0.001)	6950 (< 0.001)	30.5 (< 0.001)	−36.5	Not significant
TPC (mg GAE/100 g DW)	722 (< 0.001)	1669 (< 0.001)	37.0 (< 0.001)	+74.4	Significant
TFC (mg QE/100 g DW)	231 (< 0.001)	293 (< 0.001)	9.7 (0.014)	+7.88	Significant
TAC (mg/kg DW)[a]	2524 (< 0.001)	40.1 (< 0.001)	1.6 (0.241)	−1.04	Significant
DPPH scavenging (%)	122 (< 0.001)	457 (< 0.001)	4.0 (0.081)	−7.51	Significant
ABTS scavenging (%)	2132 (< 0.001)	2701 (< 0.001)	295 (< 0.001)	−18.0	Significant
ADH activity (%)	122 (< 0.001)	548 (< 0.001)	29.9 (< 0.001)	−5.45	Not significant
AChE inhibition (%)	94.4 (< 0.001)	612 (< 0.001)	57.4 (< 0.001)	−12.7	Significant
Specific volume (mL/g)	163 (< 0.001)	0.1 (0.737)	6.3 (0.036)	+0.40	Not significant

Crumb firmness (g)	30.9 (< 0.001)	0.2 (0.670)	10.8 (0.011)	−146.3	Significant
Staling rate (g/day)	56.2 (< 0.001)	90.7 (< 0.001)	42.0 (< 0.001)	+64.3	Significant
L*	233 (< 0.001)	55.5 (< 0.001)	2.2 (0.177)	−2.92	Significant
a*	1148 (< 0.001)	294 (< 0.001)	88.0 (< 0.001)	−2.69	Significant
b*	241 (< 0.001)	239 (< 0.001)	274 (< 0.001)	−10.3	Significant

Two-way ANOVA (general linear model) with black wheat (refined white wheat flour only vs 50:50 refined white wheat–whole black wheat flour) and co-culture fermented Cili fruit (absent vs 30% of flour weight) as fixed factors; n = 3 replicates per bread; each F has df = 1, 8. Values are F statistics with p values in parentheses. IDF, SDF and TDF refer to the breads.

[a] TAC values are not corrected for background absorbance; WB was below the limit of detection, and the signal in BsLp reflects background absorbance from the Cili ingredient (Section 2.5.2).

[b] Interaction contrast = (WWBW-BsLp − WWBW) − (BsLp − WB), i.e., the effect of fermented Cili in the composite bread minus its effect in the refined-wheat bread, in the units of each parameter. A positive value indicates that the effect of fermented Cili was more positive (or less negative) in the composite bread.

WB, refined white wheat control bread; BsLp, refined white wheat bread with 30% co-culture fermented Cili fruit; WWBW, refined white wheat-whole black wheat composite bread; WWBW-BsLp, composite bread with 30% co-culture fermented Cili fruit. IDF, SDF, TDF, insoluble, soluble and total dietary fiber; GABA, γ-aminobutyric acid; TPC, total phenolic content; TFC, total flavonoid content; TAC, total anthocyanin content; ADH, alcohol dehydrogenase; AChE, acetylcholinesterase; DW, dry weight.
